# Supplementary material for: Landscape permeability and individual variation in a dispersal‐linked gene jointly determine genetic structure in the Glanville fritillary butterfly
Source: Evol Lett. 2018 Nov 16;2(6):544–56. doi: 10.1002/evl3.90 (PMC6292703; doi:10.1002/evl3.90)
Supplement: Supplementary file 1 — Figure S1. Cluster membership estimated from BAPS for 2011 (A) and 2012 (B). Each point is a patch and the colour indicates membership to a particular cluster. Figure S2. Associations between the frequency of the Pgi c allele and two indices of connectivity (Siwater+forest and log‐transformed Simetapop) for each genetic cluster in 2011 (A) and 2012 (B). Figure S3. Q‐Q plots showing observed and expected –log10 p‐values for latent factor mixed models testing for associations between 39 loci and connectivity metrics in new populations in 2011 (A‐E) and Supporting Figures – DiLeo et al. Evolution Letters 2012 (F‐G). Figure S4. Scatterplots showing the relationship between the frequency of the Pgi‐c allele and a patch connectivity metric incorporating roads as a facilitator to dispersal (A) and water as a barrier to dispersal (B) for the year 2012. Figure S5. Scatterplots showing relationships between population‐specific genetic differentiation (Fst) and the frequency of the Pgi‐c allele (A), Fst and Siforest (B) and Fst and Siwater (C) for newly colonized populations in 2011. Figure S6. Sources of variation in patch connectivity per year. Yearly differences in connectivity for newly colonized populations, Simetapop, (A), geographic distance between source and target patches for newly colonized populations, dij (B), number of nests in source patches, Nj (C), and total number of source patches (D). Figure S7. Relationships between connectivity and minor allele frequencies (MAF) of the four other candidate loci in newly colonized and old populations. [file EVL3-2-544-s001.pdf]

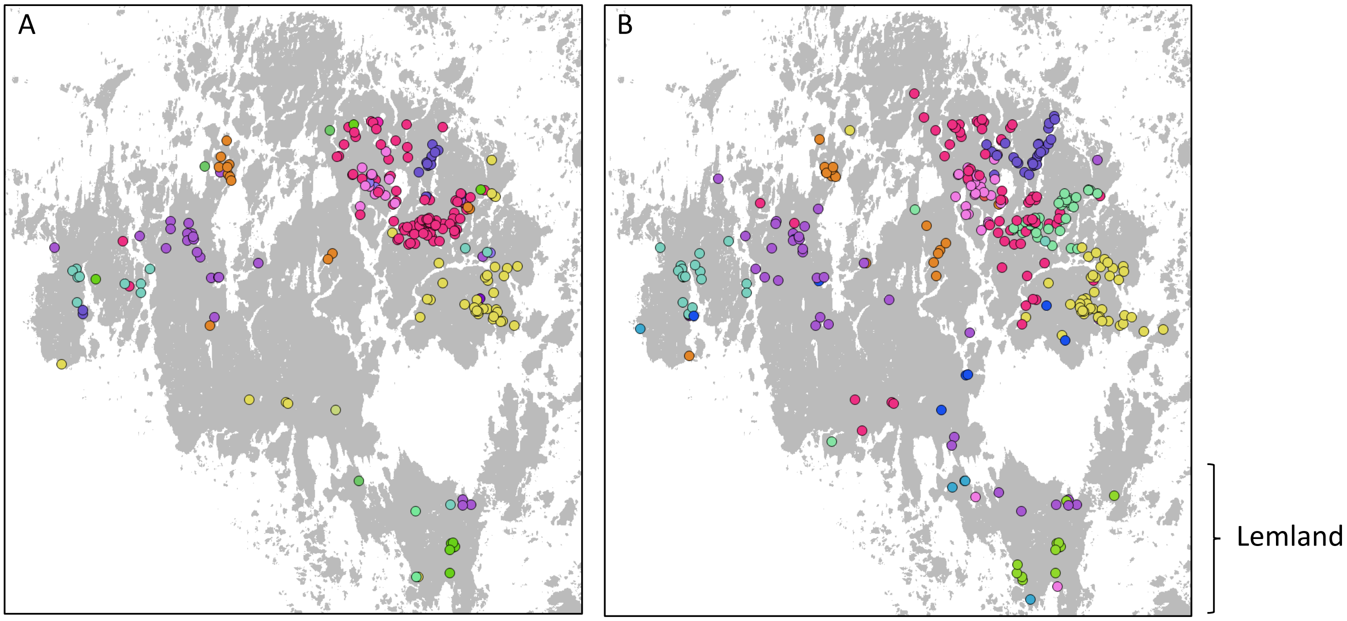

Figure S1. Cluster membership estimated from BAPS for 2011 (A) and 2012 (B). Each point is a patch and the colour indicates membership to a particular cluster. Patches in Lemland in the south of Åland were excluded from downstream analyses (see Methods in main text)

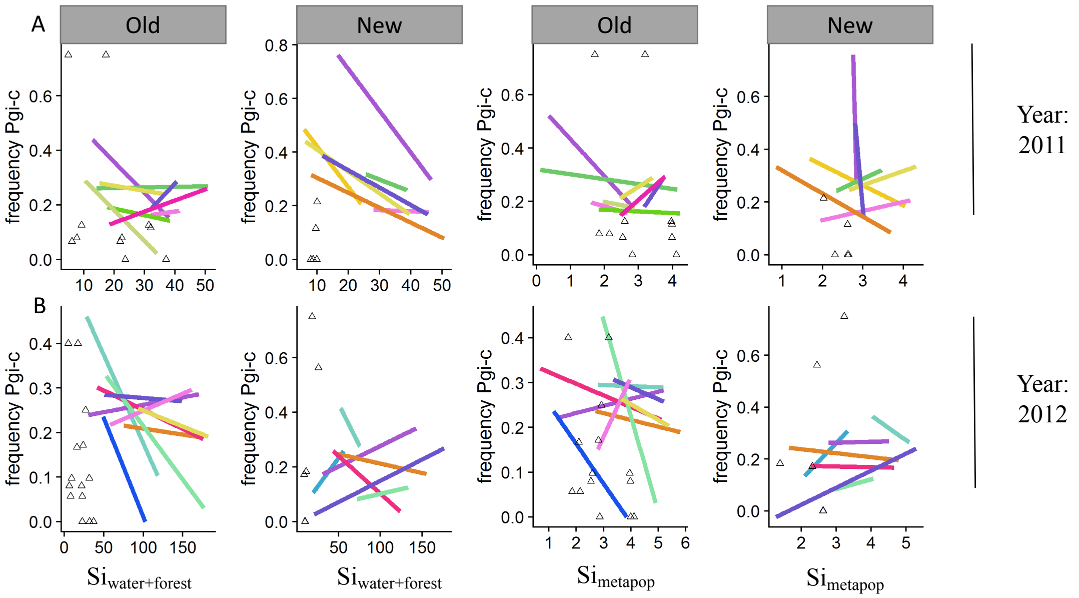

Figure S2. Associations between the frequency of the *Pgi* c allele and two indices of connectivity ($Si_{water+forest}$ and log-transformed $Si_{metapop}$) for each genetic cluster in 2011 (A) and 2012 (B). Points have been excluded for clarity, and lines are predictions from simple linear models for the purpose of showing variation in associations among the different genetic clusters. Line colour indicates genetic cluster membership, and colours match those presented in Figure S1. New and old populations are shown separately. The open triangles are populations from Lemland that were excluded from the analysis.

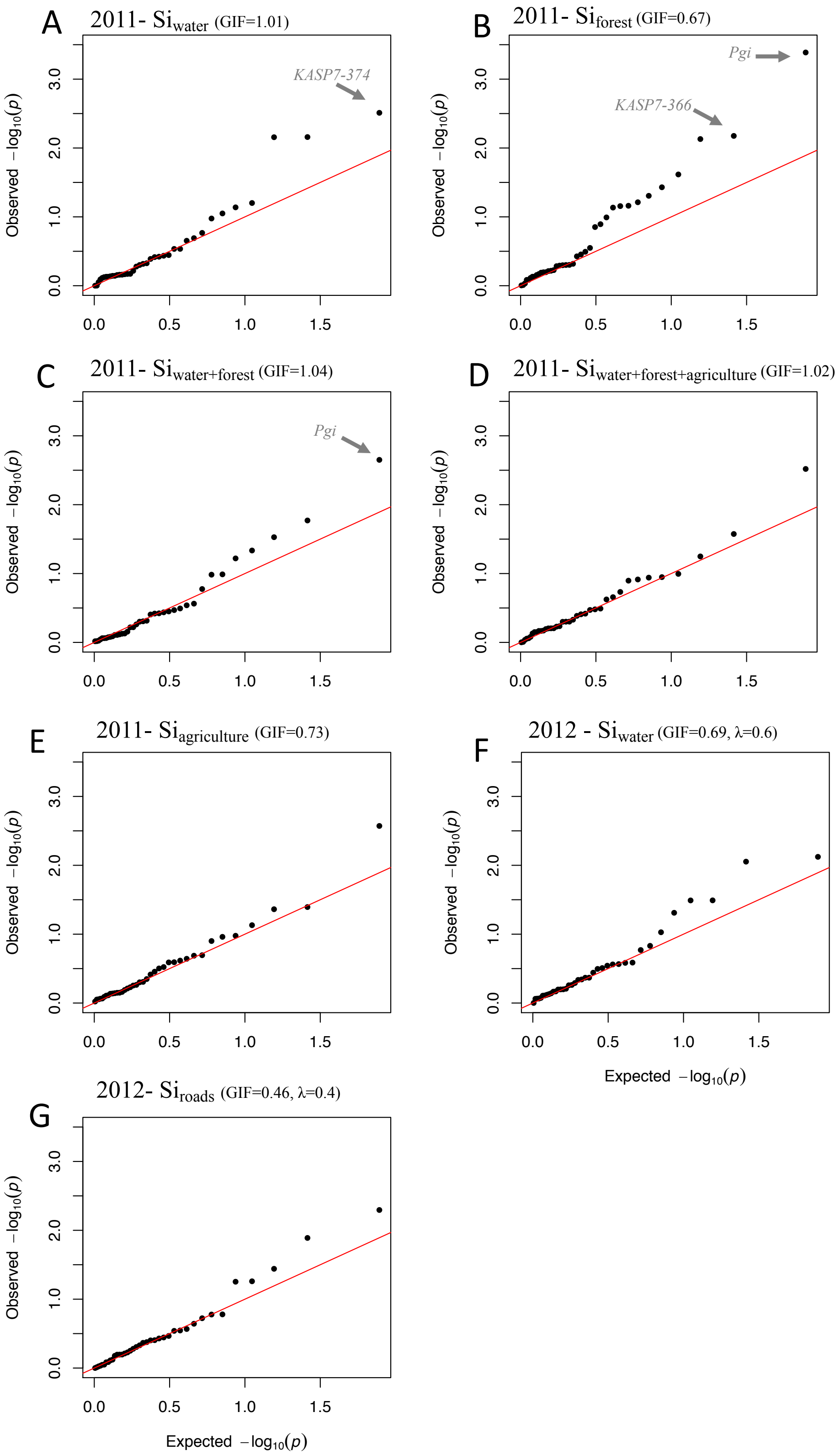

Figure S3. Q-Q plots showing observed and expected –log10 p-values for latent factor mixed models testing for associations between 39 loci and connectivity metrics in new populations in 2011 (A-E) and

2012 (F-G). The connectivity metric included as the predictor in the model is labeled in each plot. Q-Q plots are shown after *p* value calibration with either the genomic inflation factor (GIF; A-E), or λ in the case when tests were found to be overly conservative (F-G). Loci that were found to be significant at a false discovery rate of 10% are labelled with an arrow.

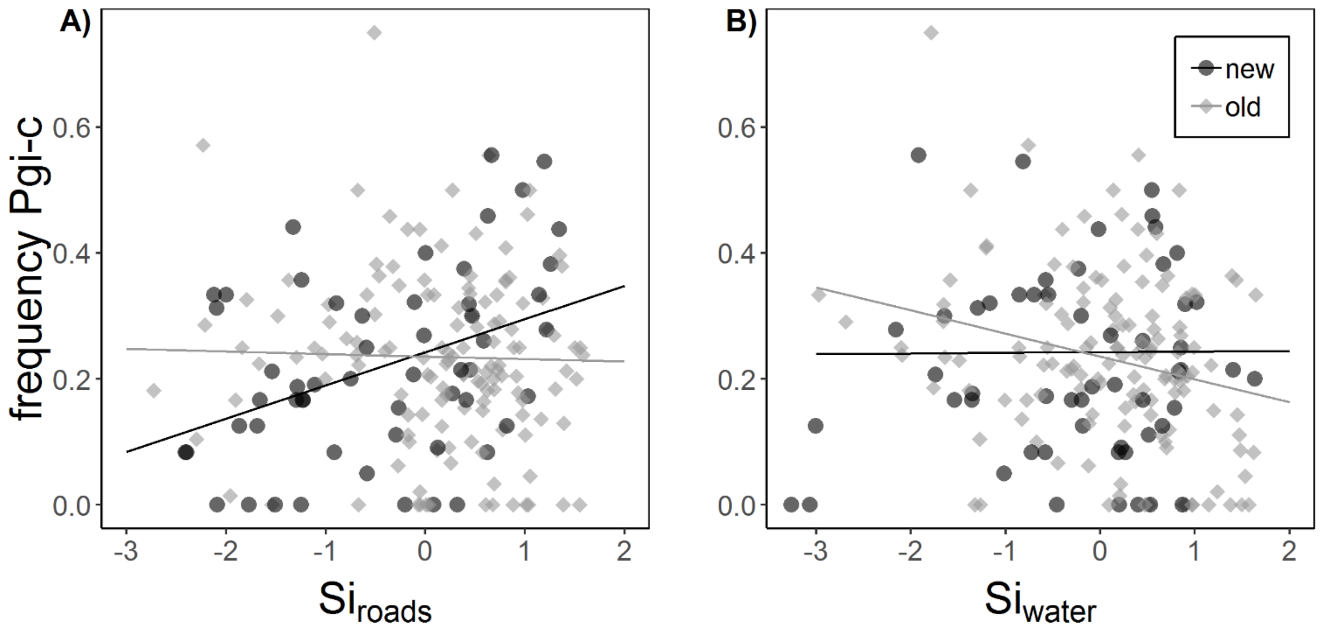

Figure S4. Scatterplots showing the relationship between the frequency of the *Pgi-c* allele and a patch connectivity metric incorporating roads as a facilitator to dispersal (A) and water as a barrier to dispersal (B) for the year 2012. Points represent local populations and old and newly colonized populations are shown in contrasting colours. Lines show predicted values of fixed effects estimated from the top supported linear mixed effect model (see Table S5 for results of model selection on this data).

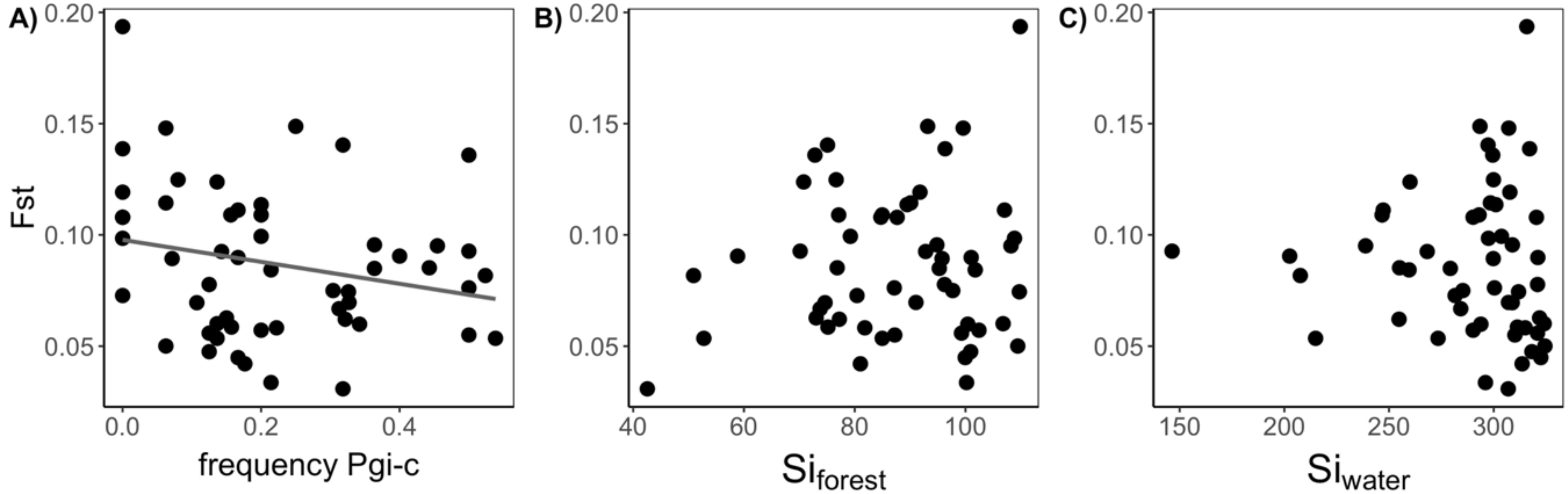

Figure S5. Scatterplots showing relationships between population-specific genetic differentiation (F_{st}) and the frequency of the *Pgi-c* allele (A), F_{st} and Si_{forest} (B) and F_{st} and Si_{water} (C) for newly colonized populations in 2011. The line shows the predicted fixed effects estimated in a linear model. No significant direct effect of connectivity on F_{st} was found.

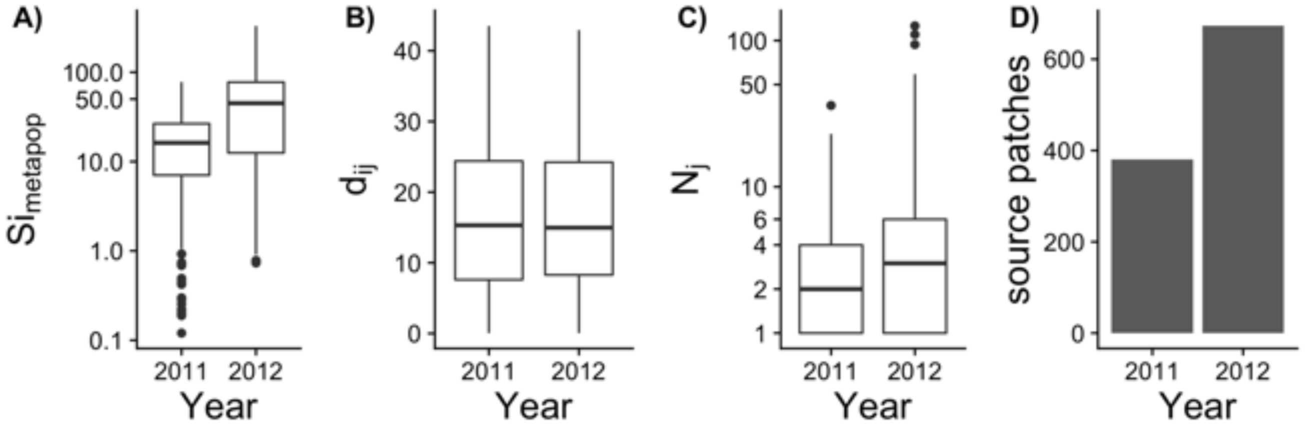

Figure S6. Sources of variation in patch connectivity per year. Yearly differences in connectivity for newly colonized populations, $Si_{metapop}$, (A), geographic distance between source and target patches for newly colonized populations, d_{ij} (B), number of nests in source patches, N_j (C), and total number of source patches (D). It can be seen that the high patch connectivity in 2012, as calculated by $S_i = \sum_{i \neq j} \exp(-\alpha d_{ij}) N_j$, is mainly driven by the higher number of potential sources patches and nests per source patch. Note that the y-axes of (A) and (C) are shown on a lognormal scale.

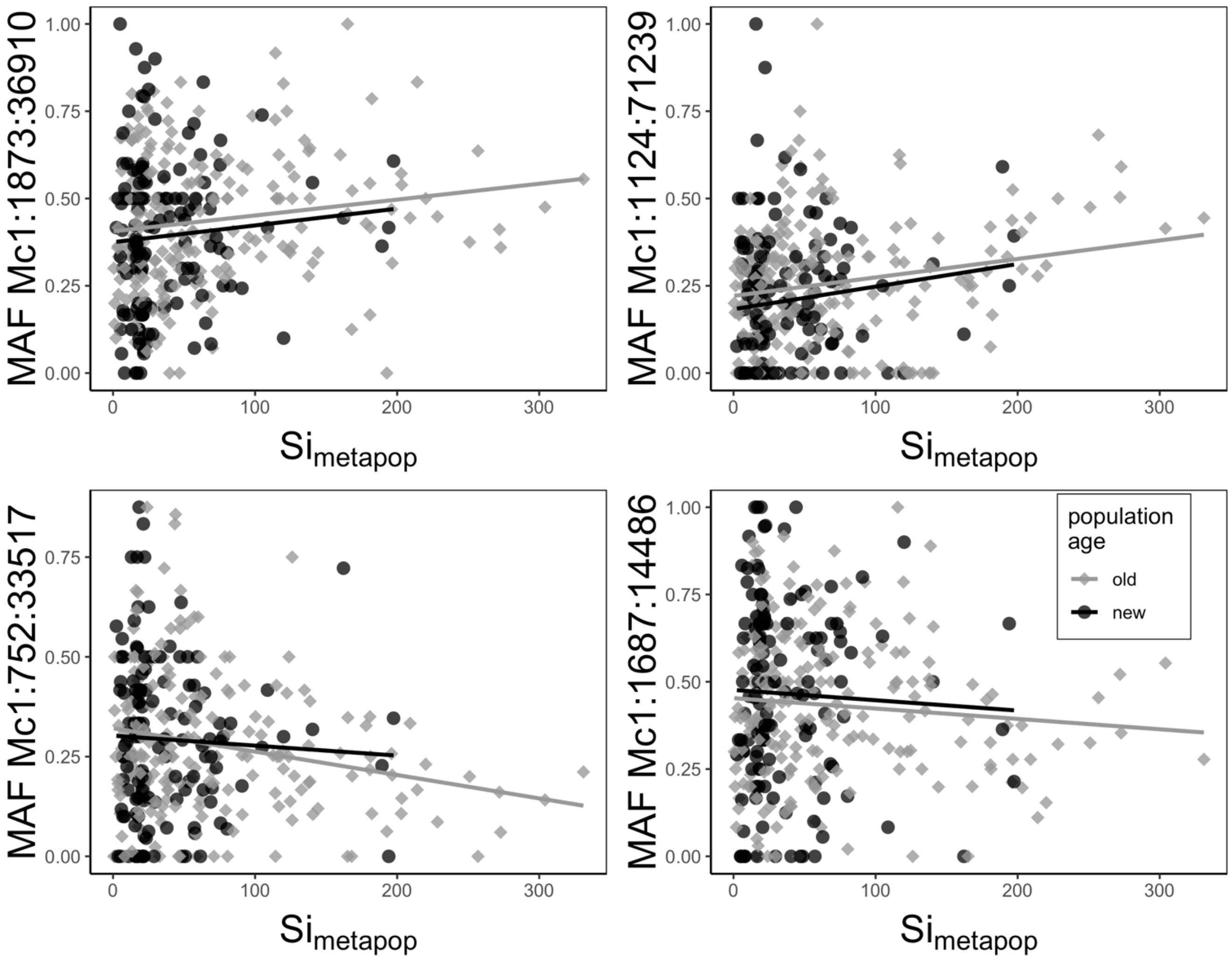

Figure S7. Relationships between connectivity and minor allele frequencies (MAF) of the four other candidate loci in newly colonized and old populations. Data from 2011 and 2012 were combined as there were little differences between the years.
