# Supplementary material for: Landscape permeability and individual variation in a dispersal‐linked gene jointly determine genetic structure in the Glanville fritillary butterfly
Source: Evol Lett. 2018 Nov 16;2(6):544–56. doi: 10.1002/evl3.90 (PMC6292703; doi:10.1002/evl3.90)
Supplement: Supplementary file 2 — Table S1. Descriptions of landscape connectivity hypotheses. Table S2. Pearson correlation coefficients among the five landscape connectivity variables. Table S3. Posterior mean estimates, standard deviation, and quantiles of Bayesian nonspatial and spatial INLA models. Table S4. Results of model selection on linear mixed effect models testing for associations between the frequency of the Pgi‐c allele, population age (old or new), and competing metrics of patch connectivity (Si) for the year 2011. Table S5. Results of model selection on linear mixed effect models testing for associations between the frequency of the Pgi‐c allele, population age (old or new), and competing metrics of patch connectivity (Si) for the year 2012. Only the top 15 models are shown. Table S6. Genomic inflation factors of for each value of k and each predictor tested in latent factor mixed models. Table S7. Results of model selection on linear mixed effect models testing for associations between population genetic differentiation (Fst), population age (old or new), and competing metrics of patch connectivity (Si) for the year 2011. Table S8. Results of model selection on linear mixed effect models testing for associations between population genetic differentiation (Fst), population age (old or new), and competing metrics of patch connectivity (Si) for the year 2011 with a singly outlier with a high Fst value removed. Only the top 15 models are shown. Table S9. Results of model selection on linear mixed effect models testing for associations between population genetic differentiation (Fst), population age (old or new), and competing metrics of patch connectivity (Si) for the year 2012. [file EVL3-2-544-s002.docx]

**Table S1.** Descriptions of landscape connectivity hypotheses. The resistance values assigned to each landscape feature for calculations of effective distance using CIRCUITSCAPE is given for each model. Model Si_metapop_ was calculated using Euclidean distances among patches and so it has no associated resistance values. The alpha value used to scale the dispersal kernel in the calculation of patch connectivity is also shown. The five models shaded in light grey were used for the main analyses. The two models shaded in dark grey were additionally used in latent factor mixed models.

**Table S2.** Pearson correlation coefficients among the five landscape connectivity variables. Correlations in 2011 are given below the diagonal and correlations in 2012 are given above the diagonal.

|  | Si_metapop_ | Si_water_ | Si_roads_ | Si_forest_ | Si_agriculture_ |
| --- | --- | --- | --- | --- | --- |
| Si_metapop_ | 1 | 0.40 | 0.59 | 0.47 | 0.01 |
| Si_water_ | 0.26 | 1 | 0.25 | 0.25 | -0.18 |
| Si_roads_ | 0.48 | 0.08 | 1 | 0.37 | -0.05 |
| Si_forest_ | 0.44 | 0.42 | 0.21 | 1 | -0.30 |
| Si_agriculture_ | 0.05 | -0.17 | -0.02 | -0.34 | 1 |

**Table S3**. Posterior mean estimates, standard deviation, and quantiles of Bayesian non-spatial and spatial INLA models. The tested models were the top models identified from model selection in 2011 (see Table S4) and 2012 (see Table S5). Note the age estimates are given as a function of their difference from the mean.

|  |  | non-spatial | | | |  | spatial | | | |
| --- | --- | --- | --- | --- | --- | --- | --- | --- | --- | --- |
| Year | Parameter | mean | sd | 0.025q | 0.975q |  | mean | sd | 0.025q | 0.975q |
| 2011 | (intercept) | 0.15 | 18.3 | -35.7 | 36.0 |  | 0.15 | 18.26 | -35.7 | 36.0 |
|  | ageOLD | 0.08 | 18.3 | -35.8 | 35.9 |  | 0.09 | 18.26 | -35.8 | 35.9 |
|  | ageNEW | 0.07 | 18.3 | -35.8 | 35.9 |  | 0.07 | 18.26 | -35.8 | 35.9 |
|  | Si_forest_ | 0.006 | 0.02 | -0.03 | 0.04 |  | -0.003 | 0.02 | -0.04 | 0.03 |
|  | Si_water_ | -0.03 | 0.01 | -0.06 | -0.009 |  | -0.02 | 0.02 | -0.06 | 0.009 |
|  | Si_agriculture_ | -0.02 | 0.01 | -0.05 | 0.004 |  | -0.02 | 0.02 | -0.05 | 0.02 |
|  | age:Si_forest_ | -0.06 | 0.02 | -0.11 | -0.02 |  | -0.06 | 0.02 | -0.10 | -0.02 |
|  |  |  |  |  |  |  |  |  |  |  |
| 2012 | (intercept) | 0.16 | 18.26 | -35.7 | 36.0 |  | 0.163 | 18.26 | -35.68 | 35.9 |
|  | ageOLD | 0.08 | 18.3 | -35.8 | 35.9 |  | 0.08 | 18.26 | -35.8 | 35.9 |
|  | ageNEW | 0.08 | 18.3 | -35.8 | 35.9 |  | 0.08 | 18.26 | -35.8 | 35.9 |
|  | Si_water_ | -0.04 | 0.01 | -0.06 | -0.01 |  | -0.01 | 0.02 | -0.04 | 0.02 |
|  | Si_roads_ | -0.004 | 0.01 | -0.03 | 0.02 |  | -0.0001 | 0.01 | -0.03 | 0.03 |
|  | age:Si_water_ | 0.04 | 0.02 | -0.004 | 0.08 |  | 0.02 | 0.02 | -0.02 | 0.06 |
|  | age:Si_roads_ | 0.06 | 0.02 | 0.01 | 0.1 |  | 0.05 | 0.02 | 0.009 | 0.09 |

**Table S4**. Results of model selection on linear mixed effect models testing for associations between the frequency of the *Pgi*-*c* allele, population age (old or new), and competing metrics of patch connectivity (Si) for the year 2011. Only the top 15 models are shown. Variances explained by fixed effects (R^2^_m_) and jointly by fixed and random effects (R^2^_c_) are shown. Models highlighted in grey were retained as a candidate set for further analysis and interpretation (see Methods). See Table 1 of main text for parameter estimates of the candidate set.

| Year | Model | R^2^_m_ | R^2^_c_ | AICc | ΔAICc |
| --- | --- | --- | --- | --- | --- |
| 2011 | age*Si_forest_ + Si_water_ + Si_agriculture_ | 0.15 | 0.18 | -148.5 | 0 |
|  | age*Si_forest_ + age*Si_water_ + Si_agriculture_ | 0.16 | 0.20 | -148.3 | 0.2 |
|  | age*Si_forest_ + Si_water_ | 0.13 | 0.16 | -147.7 | 0.8 |
|  | age*Si_forest_ + Si_water_ + Si_agriculture_ + Si_roads_ | 0.15 | 0.19 | -147.2 | 1.3 |
|  | age*Si_forest_ + age*Si_roads_ + Si_water_ + Si_agriculture_ | 0.17 | 0.20 | -147.1 | 1.4 |
|  | age*Si_forest_ + age*Si_water_ | 0.14 | 0.17 | -146.8 | 1.7 |
|  | age*Si_forest_ + age*Si_water_ + age*Si_roads_ + Si_agriculture_ | 0.17 | 0.21 | -146.8 | 1.7 |
|  | age*Si_water_ + Si_forest_ + Si_agriculture_ | 0.14 | 0.17 | -146.5 | 2.0 |
|  | age*Si_forest_ + age*Si_agriculture_ + Si_water_ | 0.15 | 0.18 | -146.5 | 2.0 |
|  | age*Si_water_ + age*Si_roads_ + Si_forest_ + Si_agriculture_ | 0.16 | 0.20 | -146.5 | 2.0 |
|  | age*Si_forest_ + age*Si_water_ + Si_roads_ + Si_agriculture_ | 0.16 | 0.20 | -146.4 | 2.1 |
|  | age*Si_forest_ + age*Si_water_ + age*Si_agriculture_ | 0.16 | 0.20 | -146.3 | 2.2 |
|  | age*Si_forest_ + Si_water_ + Si_agriculture_ + Si_metapop_ | 0.15 | 0.18 | -146.3 | 2.2 |
|  | age*Si_forest_ + Si_water_ + Si_roads_ | 0.14 | 0.17 | -146.2 | 2.3 |
|  | age*Si_forest_ + age*Si_water_ + Si_agriculture_ + Si_metapop_ | 0.16 | 0.20 | -146.1 | 2.4 |
|  |  |  |  |  |  |

**Table S5.** Results of model selection on linear mixed effect models testing for associations between the frequency of the *Pgi*-*c* allele, population age (old or new), and competing metrics of patch connectivity (Si) for the year 2012. Only the top 15 models are shown. Variances explained by fixed effects (R^2^_m_) and jointly by fixed and random effects (R^2^_c_) are shown. Models highlighted in grey were retained as a candidate set for further analysis and interpretation (see Methods). See Table 1 of main text for parameter estimates of the candidate set.

| Year | Model | R^2^_m_ | R^2^_c_ | AICc | ΔAICc |
| --- | --- | --- | --- | --- | --- |
| 2012 | age*Si_roads_ + age*Si_water_ | 0.10 | 0.10 | -216.4 | 0 |
|  | age*Si_roads_ + age*Si_water_ + Si_agriculture_ | 0.10 | 0.11 | -215.3 | 1.1 |
|  | age*Si_roads_ + Si_water_ | 0.08 | 0.09 | -215.2 | 1.2 |
|  | age*Si_roads_ + age*Si_water_ + Si_metapop_ | 0.10 | 0.10 | -215 | 1.4 |
|  | age*Si_roads_ + age*Si_water_ + Si_forest_ | 0.10 | 0.10 | -214.9 | 1.5 |
|  | age*Si_roads_ + age*Si_water_ + Si_forest_  + Si_agriculture_ + Si_metapop_ | 0.11 | 0.11 | -214.5 | 1.9 |
|  | age*Si_roads_ + Si_water_ + Si_agriculture_ | 0.08 | 0.09 | -213.8 | 2.6 |
|  | age*Si_roads_ + age*Si_water_ + Si_agriculture_ + Si_metapop_ | 0.11 | 0.11 | -213.7 | 2.7 |
|  | age*Si_roads_ + Si_water_ + Si_forest_ | 0.08 | 0.09 | -213.7 | 2.7 |
|  | age*Si_roads_ + age*Si_water_ + age*Si_agriculture_ | 0.11 | 0.11 | -213.7 | 2.7 |
|  | age*Si_roads_ + Si_water_ + Si_metapop_ | 0.08 | 0.09 | -213.5 | 2.9 |
|  | age*Si_roads_ + age*Si_water_ + age*Si_agriculture_ + Si_forest_ | 0.12 | 0.12 | -213.2 | 3.2 |
|  | age*Si_roads_ + age*Si_water_ + Si_forest_  + Si_metapop_ | 0.10 | 0.10 | -213.2 | 3.2 |
|  | age*Si_roads_ | 0.05 | 0.08 | -213 | 3.4 |
|  | age*Si_roads_ + age*Si_water_ + age*Si_forest_ | 0.10 | 0.10 | -212.9 | 3.5 |

**Table S6.** Genomic inflation factors of for each value of *k* and each predictor tested in latent factor mixed models. The value of *k* reported in the results correspond to the rows shaded in grey for each predictor.

| *k* | 2011 | | | | |  | 2012 | |
| --- | --- | --- | --- | --- | --- | --- | --- | --- |
|  | Si_water_ | Si_water+forest_ | Si_water+forest+agri_ | Si_forest_ | Si_agriculture_ |  | Si_water_ | Si_roads_ |
| 7 | 1.27 | 1.41 | 1.14 | 0.67 | 0.73 |  | 0.69 | 0.46 |
| 8 | 1.23 | 1.31 | 1.02 | 0.63 | 0.65 |  | 0.54 | 0.42 |
| 9 | 1.2 | 1.28 | 0.93 | 0.63 | 0.55 |  | 0.48 | 0.39 |
| 10 | 1.19 | 1.22 | 0.83 | 0.58 | 0.54 |  | 0.48 | 0.32 |
| 11 | 1.57 | 1.2 | 0.83 | 0.56 | 0.53 |  | 0.43 | 0.32 |
| 12 | 1.16 | 1.2 | 0.83 | 0.53 | 0.51 |  | 0.43 | 0.28 |
| 13 | 1.07 | 1.1 | 0.75 | 0.53 | 0.48 |  | 0.31 | 0.19 |
| 14 | 1.01 | 1.1 | 0.67 | 0.51 | 0.49 |  | 0.04 | 0.04 |
| 15 | 1.03 | 1.04 | 0.65 | 0.48 | 0.46 |  | 0.03 | 0.018 |

**Table S7.** Results of model selection on linear mixed effect models testing for associations between population genetic differentiation (F_st_), population age (old or new), and competing metrics of patch connectivity (Si) for the year 2011. Only the top 15 models are shown. Variances explained by fixed effects (R^2^_m_) and jointly by fixed and random effects (R^2^_c_) are shown.

| Year | Model | R^2^_m_ | R^2^_c_ | AICc | ΔAICc |
| --- | --- | --- | --- | --- | --- |
| 2011 | intercept only |  | 0.02 | -464.5 | 0 |
|  | Si_water_ + Si_forest_ | 0.04 | 0.04 | -464.4 | 0.1 |
|  | Si_water_ | 0.02 | 0.02 | -464 | 0.5 |
|  | Si_water_ + Si_forest_ + Si_agriculture_ | 0.05 | 0.05 | -463.7 | 0.8 |
|  | age | 0.01 | 0.04 | -463.4 | 1.1 |
|  | Si_forest_ | 0.01 | 0.04 | -463.4 | 1.1 |
|  | age + Si_water_ + Si_forest_ | 0.05 | 0.07 | -463.3 | 1.2 |
|  | Si_agriculture_ | 0.005 | 0.02 | -462.9 | 1.6 |
|  | age + Si_water_ + Si_forest_ + Si_agriculture_ | 0.07 | 0.07 | -462.8 | 1.7 |
|  | age + Si_water_ | 0.02 | 0.04 | -462.8 | 1.7 |
|  | Si_roads_ | 0.003 | 0.03 | -462.6 | 1.9 |
|  | Si_metapop_ | 0.001 | 0.02 | -462.5 | 2.0 |
|  | Si_metapop_ + Si_water_ | 0.02 | 0.02 | -462.5 | 2.0 |
|  | Si_forest_ + Si_agriculture_ | 0.02 | 0.05 | -462.4 | 2.1 |
|  | age + Si_forest_ | 0.02 | 0.06 | -462.4 | 2.1 |

**Table S8.** Results of model selection on linear mixed effect models testing for associations between population genetic differentiation (F_st_), population age (old or new), and competing metrics of patch connectivity (Si) for the year 2011 with a singly outlier with a high F_st_ value removed. Only the top 15 models are shown. Variances explained by fixed effects (R^2^_m_) and jointly by fixed and random effects (R^2^_c_) are shown.

| Year | Model | R^2^_m_ | R^2^_c_ | AICc | ΔAICc |
| --- | --- | --- | --- | --- | --- |
| 2011, outlier removed | Si_water_ | 0.03 | 0.03 | -476.8 | 0 |
|  | Si_water_ + Si_forest_ | 0.04 | 0.04 | -476 | 0.8 |
|  | Si_water_ + Si_forest_ + Si_agriculture_ | 0.06 | 0.06 | -475.5 | 1.3 |
|  | intercept only |  | 0.003 | -475.5 | 1.3 |
|  | Si_water_ + Si_agriculture_ | 0.04 | 0.04 | -475.4 | 1.4 |
|  | age + Si_water_ | 0.04 | 0.04 | -475 | 1.8 |
|  | Si_water_ + Si_metapop_ | 0.03 | 0.03 | -474.8 | 2.0 |
|  | Si_water_ + Si_roads_ | 0.03 | 0.03 | -474.6 | 2.2 |
|  | Si_agriculture_ | 0.01 | 0.01 | -474.4 | 2.4 |
|  | age + Si_water_ + Si_forest_ | 0.05 | 0.05 | -474.2 | 2.5 |
|  | age + Si_water_ + Si_forest_ + Si_agriculture_ | 0.07 | 0.07 | -474 | 2.8 |
|  | Si_water_ + Si_forest_ + Si_roads_ | 0.05 | 0.05 | -473.8 | 3.0 |
|  | Si_water_ + Si_forest_ + Si_metapop_ | 0.04 | 0.04 | -473.8 | 3.0 |
|  | age + Si_water_ + Si_agriculture_ | 0.04 | 0.04 | -473.8 | 3.0 |
|  | age | 0.004 | 0.01 | -473.7 | 3.1 |

**Table S9**. Results of model selection on linear mixed effect models testing for associations between population genetic differentiation (F_st_), population age (old or new), and competing metrics of patch connectivity (Si) for the year 2012. Only the top 15 models are shown. Variances explained by fixed effects (R^2^_m_) and jointly by fixed and random effects (R^2^_c_) are shown. Models highlighted in grey were retained as a candidate set for further interpretation (see Methods). See Table 2 of main text for parameter estimates of the candidate set.

| Year | Model | R^2^_m_ | R^2^_c_ | AICc | ΔAICc |
| --- | --- | --- | --- | --- | --- |
| 2012 | Si_metapop_ + Si_water_ | 0.11 | 0.16 | -713.3 | 0 |
|  | Si_metapop_ + Si_water_ + Si_roads_ | 0.11 | 0.17 | -712.4 | 0.9 |
|  | Si_metapop_ + Si_water_ + Si_agriculture_ | 0.11 | 0.17 | -712.3 | 0.9 |
|  | Si_metapop_ | 0.08 | 0.16 | -712.2 | 1.1 |
|  | Si_metapop_ + Si_agriculture_ | 0.09 | 0.17 | -712 | 1.2 |
|  | age + Si_metapop_ + Si_water_ | 0.11 | 0.17 | -711.5 | 1.8 |
|  | Si_water_ + Si_roads_ | 0.10 | 0.14 | -711.4 | 1.8 |
|  | Si_metapop_ + Si_water_+ Si_agriculture_ + Si_roads_ | 0.12 | 0.17 | -711.2 | 2.0 |
|  | Si_metapop_+ Si_roads_ | 0.09 | 0.17 | -711.2 | 2.1 |
|  | Si_metapop_ + Si_water_ + Si_forest_ | 0.11 | 0.16 | -711.1 | 2.2 |
|  | Si_metapop_ + Si_agriculture_ + Si_roads_ | 0.10 | 0.17 | -710.8 | 2.4 |
|  | age + Si_metapop_ + Si_water_ + Si_roads_ | 0.12 | 0.17 | -710.7 | 2.6 |
|  | age + Si_metapop_ + Si_water_ + Si_agriculture_ | 0.11 | 0.17 | -710.6 | 2.7 |
|  | Si_metapop_ + Si_water_ + Si_forest_ + Si_agriculture_ | 0.11 | 0.17 | -710.2 | 3.0 |
|  | age + Si_metapop_ | 0.09 | 0.17 | -710.2 | 3.0 |
