# Supplementary material for: Landscape permeability and individual variation in a dispersal‐linked gene jointly determine genetic structure in the Glanville fritillary butterfly
Source: Evol Lett. 2018 Nov 16;2(6):544–56. doi: 10.1002/evl3.90 (PMC6292703; doi:10.1002/evl3.90)
Supplement: Supplementary file 3 — Appendix A – Supplementary methods and results [file EVL3-2-544-s003.pdf]

Appendix A – Supplementary methods and results

SNP Genotyping

Larval tissue was homogenized and DNA was extracted as described in Fountain *et al.* (2018). Genotyping was performed on the KASP platform. In total 40 putatively neutral markers from noncoding regions of the genome were selected, and an additional five putatively functional markers were selected from candidate genes related to flight or dispersal traits including *Pgi* (Orsini *et al.* 2009; Kvist *et al.* 2013; Kvist *et al.* 2015; Wong *et al.* 2016; Duplouy *et al.* 2017; Appendix B). The neutral loci were obtained from SOLiD matepair-1 genome sequences (Ahola *et al.* 2014) using an in-house SNP calling method (Rastas *et al.* 2013). The neutral SNPs have minor allele frequencies >0.2, and span all 31 chromosomes from non-coding regions. Further details of SNP calling, validation, and quality control are described in Fountain *et al.* (2016). Previous work demonstrated that this neutral SNP panel was sufficient to capture both large (Nair *et al.* 2016) and small-scale (Fountain *et al.* 2018) patterns of genetic population structure. In a study conducted in a smaller region in Åland, Fountain *et al.* (2018) showed that the neutral 40-SNP panel and a larger panel of 272 SNPs resolved the same patterns of spatial genetic structure in each of the six years tested. We are thus confident that the panel used here will represent the genetic structure of the metapopulation well.

Development of landscape connectivity hypotheses

The landscape was classified from 20 m resolution CORINE 2012 Land Cover Inventory raster data from the Finnish Environmental Institute (http://www.syke.fi/en-US/Open_information/Spatial_datasets). The original raster layer included 48 land cover categories, 42 of which were found in our study region. We additionally overlaid polygons representing patches, and added a category representing edges between agriculture and forested areas as previous work found that

M. cinxia tend to move along edges (Ovaskainen *et al.* 2008). We simplified the final land classification by combining structurally similar landscape features, resulting in 11 distinct categories representing major land use in the study region: discontinuous urban, continuous urban, open water, closed forest, transitional woodland scrub, agriculture with no major natural elements, agricultural edges, pasture, bare rock, roads, and patches.

BAPS and latent factor mixed modeling

Latent factor mixed models (lfmm) require the number of genetically distinct populations, *k*, to be determined *apriori*. We used the Bayesian clustering program BAPS6 (Corander, Siren, and Arjas 2008, Cheng et al. 2013) to estimate *k* for *M. cinxia* in Åland in the years 2011 and 2012 separately. This program has been successfully used to quantify the number of genetically distinct populations in Åland previously (Nair et al. 2016, Orsini et al. 2008). We used the spatial mixture model, which is suggested for populations that exhibit low amounts of genetic structure or when few markers are used. Twenty replicate runs with an upper bound of 30 clusters was used. BAPS identified 15 clusters with the highest marginal likelihood in 2011 and 12 clusters in 2012 (Fig. S1). Because latent factor mixed models were run only on new populations, the actual number of clusters represented in the data were fewer – e.g. individuals from new populations with Lemland excluded were found in 10 of the 15 clusters in 2011 and 10 of the 12 clusters in 2012, but many of the clusters only included a single represented individual. To be sure, we explored a broad range of *k* values (7-15) for the latent factor mixed models. We ran the lfmms 10 times for each connectivity predictor, for 20,000 iterations following 10,000 iterations of burn-in. We calculated median z-scores across the 10 repetitions. The resulting *p*-values were calibrated to correct for type I errors by applying an inflation factor (Francois *et al.* 2016). We corrected for multiple testing by applying the Benjamini-Hochberg algorithm (Benjamini and Hochberg 1995) with a false discovery rate of 10%. After each run of the lfmm we calculated the

genomic inflation factor (GIF) as the median of the squared z-scores divided by the median of the chi-squared distribution (Francois et al. 2016). The values of the GIF can be used to evaluate how well the test captured neutral genetic variation (Francois et al. 2016). A GIF of one indicates that neutral genetic structure is controlled for properly, while a low value indicates a conservative test and a high value indicates a liberal test (Francois et al. 2016). We used the GIFs to rescale the test statistic, which is recommended to limit inflation due to confounding factors and population structure (Francois et al. 2016). Tests of Si_{water}, $Si_{water+forest}$, and $Si_{water+forest+agriculture}$ in 2011 had GIFs very close to one (Table S6). Tests of Si_{forest} and $Si_{agriculture}$ in 2011 and Si_{water} and Si_{roads} in 2012 were below one, indicated a conservative test. We found that the tests of Si_{water} and Si_{roads} were overly conservative even after calibrating p-values with the GIF, and thus applied slightly lower inflation factors to achieve a better distribution of the Q-Q plots (Fig S_lfmm). This did not change our result – no locus was identified as a significant outlier in 2012.

Spatial autocorrelation analysis

We tested the residuals of the top supported *Pgi* linear mixed effect models in 2011 and 2012 for spatial autocorrelation by plotting semivariance over distances up to 10 km at 1 km intervals. We found evidence for spatial autocorrelation up to 2-3 km in 2011 and 4-5 km in 2012. To test if this biased our results, we compared models with and without spatial random effects using Integrated Nested Laplace Approximation implemented in r-inla (Rue *et al.* 2009; Lindgren & Rue 2015). For both years, SPDE models were implemented and a 2-dimensional mesh was constructed using a cut-off of 30 metres (minimum distance between sampled patches), maximum edges of 1.5 km (inner) and 3 km (outer), and an offset of 5 km to ensure no boundary effects. The resulting number of mesh vertices were n=2150 in 2011 and n=2456 in 2012. Two models were constructed for each year; one including fixed effects

(age*Si_{forest} + Si_{water} + $Si_{agriculture}$ in 2011; age*Si_{roads} + age*Si_{water} in 2012) and a random effect of genetic cluster, and another that was identical to the first but with the addition of the spatial random effect. It was clear from the DIC values that the spatial model was preferred in both cases, however, there was very little difference in the fixed estimates generated by the spatial and non-spatial models and our conclusions did not change (Table S3). We thus chose to keep the main analysis in the text without the addition of spatial random effects.

Cheng, L., T. R. Connor, J. Siren, D. M. Aanensen, and J. Corander. (2013). Hierarchical and Spatially Explicit Clustering of DNA Sequences with BAPS Software. *Molecular Biology and Evolution,* 30:1224-1228.

Corander, J., J. Siren, and E. Arjas. (2008). "Bayesian spatial modeling of genetic population structure." *Computational Statistics,* 23:111-129.

Francois, O., H. Martins, K. Caye, and S. D. Schoville. (2016). Controlling false discoveries in genome scans for selection. *Molecular Ecology,* 25:454-469.

Lindgren, F., Rue, H. (2015). Bayesian spatial modelling with R-INLA. *Journal of Statistical Software,* 63(19).

Nair, A., T. Fountain, S. Ikonen, S. P. Ojanen, and S. van Nouhuys. (2016). Spatial and temporal genetic structure at the fourth trophic level in a fragmented landscape. *Proceedings of the Royal Society B-Biological Sciences,* 283(1831).

Orsini, L., J. Corander, A. Alasentie, and I. Hanski. (2008). Genetic spatial structure in a butterfly metapopulation correlates better with past than present demographic structure. *Molecular Ecology,* 17:2629-2642.

Rue, H., Martino, S., Chopin, N. (2009). Approximate Bayesian inference for latent Gaussian models by using integrated nested Laplace approximations. *Journal of the Royal Statistical Society Series B,* 71:319-92.
