# Supplementary material for: Landscape permeability and individual variation in a dispersal‐linked gene jointly determine genetic structure in the Glanville fritillary butterfly
Source: Evol Lett. 2018 Nov 16;2(6):544–56. doi: 10.1002/evl3.90 (PMC6292703; doi:10.1002/evl3.90)
Supplement: Supplementary file 4 — Appendix B: Information about the 5 candidate and 40 neutral SNPs used. [file EVL3-2-544-s004.docx]

Appendix B: Information about the 5 candidate and 40 neutral SNPs used. Information on the marker type (candidate or neutral), location in the genome, and annotation are given. Reference to experiments and association studies are given for the candidate loci. Neutral loci shaded in gray were excluded from analysis due to low call rates.

| SNP | KASP ID | Scaffold | Position | Gene ID in Ensembl Metazoa | Chromosome | Type | Annotation | Reference |
| --- | --- | --- | --- | --- | --- | --- | --- | --- |
| Mc1:1687:14486 | KASP2-82 | scaffold1687 | 14486 | MCINX003215 | 1 | Candidate | Flightin | Kvist et al. 2015; de Jong & Saastamoinen 2018 |
| Mc1:3283:19949 | KASP2-88 | scaffold3283 | 19949 | MCINX009374 | 25 | Candidate | Glucose-6-phosphate isomerase (Fragment) | Orsini et al. 2008; Niitepold et al. 2009; Hanski et al. 2017; Duplouy et al. 2017; Wong et al. 2016 |
| Mc1:1873:36910 | KASP3-52 | scaffold1873 | 36910 | MCINX004285 | 21 | Candidate | Dnajc30 protein, DnaJ-like protein 11 | Ahola et al. 2014; Kvist et al. 2015; Fountain et al. 2016; Duplouy et al. 2017 |
| Mc1:1124:71239 | KASP3-9 | scaffold1124 | 71239 | MCINX000453 | 11 | Candidate | XPA-binding protein 1 | Kvist et al. 2015; Fountain et al. 2016; Duplouy et al. 2017 |
| Mc1:752:33517 | KASP5-118 | scaffold752 | 33517 | MCINX014859 | 15 | Candidate | Sterol regulatory element-binding protein 1, Putative sterol regulatory element-binding protein 1 | Kvist et al. 2015; Fountain et al. 2016; Duplouy et al. 2017 |
| Mc1:2086:49305 | KASP7-112 | scaffold2086 | 49305 |  | 2 | Neutral | Not annotated |  |
| Mc1:2089:90213 | KASP7-113 | scaffold2089 | 90213 |  | 15 | Neutral | Not annotated |  |
| Mc1:2424:16092 | KASP7-127 | scaffold2424 | 16092 |  | 18 | Neutral | Not annotated |  |
| Mc1:2497:108837 | KASP7-145 | scaffold2497 | 108837 |  | 26 | Neutral | Not annotated |  |
| Mc1:1067:20731 | KASP7-16 | scaffold1067 | 20731 |  | 12 | Neutral | Not annotated |  |
| Mc1:260:88657 | KASP7-162 | scaffold260 | 88657 |  | 20 | Neutral | Not annotated |  |
| Mc1:2615:52983 | KASP7-164 | scaffold2615 | 52983 |  | 21 | Neutral | Not annotated |  |
| Mc1:2817:8307 | KASP7-185 | scaffold2817 | 8307 |  | 9 | Neutral | Not annotated |  |
| Mc1:2850:40949 | KASP7-202 | scaffold2850 | 40949 |  | 6 | Neutral | Not annotated |  |
| Mc1:2857:61593 | KASP7-212 | scaffold2857 | 61593 |  | 19 | Neutral | Not annotated |  |
| Mc1:3039:6076 | KASP7-258 | scaffold3039 | 6076 |  | 24 | Neutral | Not annotated |  |
| Mc1:1216:17862 | KASP7-27 | scaffold1216 | 17862 |  | 31 | Neutral | Not annotated |  |
| Mc1:3200:6709 | KASP7-277 | scaffold3200 | 6709 |  | 26 | Neutral | Not annotated |  |
| Mc1:3280:10986 | KASP7-283 | scaffold3280 | 10986 |  | 28 | Neutral | Not annotated |  |
| Mc1:3563:36552 | KASP7-310 | scaffold3563 | 36552 |  | 13 | Neutral | Not annotated |  |
| Mc1:3659:48449 | KASP7-315 | scaffold3659 | 48449 |  | 13 | Neutral | Not annotated |  |
| Mc1:3685:41884 | KASP7-321 | scaffold3685 | 41884 |  | 28 | Neutral | Not annotated |  |
| Mc1:3693:14961 | KASP7-324 | scaffold3693 | 14961 |  | 30 | Neutral | Not annotated |  |
| Mc1:382:81301 | KASP7-337 | scaffold382 | 81301 |  | 10 | Neutral | Not annotated |  |
| Mc1:3859:67273 | KASP7-345 | scaffold3859 | 67273 |  | 11 | Neutral | Not annotated |  |
| Mc1:4270:52086 | KASP7-360 | scaffold4270 | 52086 |  | 16 | Neutral | Not annotated |  |
| Mc1:4286:9342 | KASP7-361 | scaffold4286 | 9342 |  | 16 | Neutral | Not annotated |  |
| Mc1:4405:11378 | KASP7-366 | scaffold4405 | 11378 |  | 27 | Neutral | Not annotated |  |
| Mc1:444:104441 | KASP7-374 | scaffold444 | 104441 |  | 22 | Neutral | Not annotated |  |
| Mc1:4661:252 | KASP7-389 | scaffold4661 | 252 |  | 29 | Neutral | Not annotated |  |
| Mc1:5338:8223 | KASP7-454 | scaffold5338 | 8223 |  | 19 | Neutral | Not annotated |  |
| Mc1:5357:12554 | KASP7-456 | scaffold5357 | 12554 |  | 23 | Neutral | Not annotated |  |
| Mc1:546:53771 | KASP7-467 | scaffold546 | 53771 |  | 17 | Neutral | Not annotated |  |
| Mc1:552:117110 | KASP7-469 | scaffold552 | 117110 |  | 12 | Neutral | Not annotated |  |
| Mc1:70:16568 | KASP7-486 | scaffold70 | 16568 |  | 1 | Neutral | Not annotated |  |
| Mc1:7393:34989 | KASP7-488 | scaffold7393 | 34989 |  | 7 | Neutral | Not annotated |  |
| Mc1:7875:6448 | KASP7-499 | scaffold7875 | 64448 |  | 14 | Neutral | Not annotated |  |
| Mc1:845:43108 | KASP7-512 | scaffold835 | 43108 |  | 3 | Neutral | Not annotated |  |
| Mc1:9635:49906 | KASP7-527 | scaffold9635 | 4996 |  | 3 | Neutral | Not annotated |  |
| Mc1:1337:10523 | KASP7-53 | scaffold1337 | 10523 |  | 4 | Neutral | Not annotated |  |
| Mc1:1777:7191 | KASP7-76 | scaffold1777 | 7191 |  | 1 | Neutral | Not annotated |  |
| Mc1:1799:11328 | KASP7-81 | scaffold1799 | 11328 |  | 25 | Neutral | Not annotated |  |
| Mc1:1810:50056 | KASP7-83 | scaffold1810 | 50056 |  | 11 | Neutral | Not annotated |  |
| Mc1:1826:3522 | KASP7-84 | scaffold1826 | 3522 |  | 22 | Neutral | Not annotated |  |
| Mc1:2003:17223 | KASP7-97 | scaffold2003 | 17223 |  | 6 | Neutral | Not annotated |  |
